# Molecular Diagnosis of Neurofibromatosis by Multigene Panel Testing

**DOI:** 10.3389/fgene.2021.603195

**Published:** 2021-03-09

**Authors:** Zeng-Yun-Ou Zhang, Yuan-Yuan Wu, Xin-ying Cai, Wen-Liang Fang, Feng-Li Xiao

**Affiliations:** ^1^Department of Dermatology, First Affiliated Hospital of Anhui Medical University, Hefei, China; ^2^Key Laboratory of Dermatology, Ministry of Education, Anhui Medical University, Hefei, China; ^3^Clinical College, Anhui Medical University, Hefei, China; ^4^The Center for Scientific Research of Anhui Medical University, Hefei, China

**Keywords:** neurofibromatosis type 1⋅NF1, neurofibromatosis type 2⋅NF2, mosaic neurofibromatosis type 1⋅MNF1, multi-gene panel testing, ultra-deep sequencing

## Abstract

Neurofibromatosis (NF) is an autosomal genetic disorder for which early and definite clinical diagnoses are difficult. To identify the diagnosis, five affected probands with suspected NF from unrelated families were included in this study. Molecular analysis was performed using multigene panel testing and Sanger sequencing. Ultradeep sequencing was used to analyze the mutation rate in the tissues from the proband with mosaic mutations. Three different pathogenic variants of the *NF1* gene were found in three probands who mainly complained of café-au-lait macules (CALMs), including one frameshift variant c.5072_5073insTATAACTGTAACTCCTGGGTCAGGGAGTACACCAA:p.Tyr1692Ilefs in exon 37, one missense variant c.3826C > T:p.Arg1276Ter in exon 28, and one splicing variant c.4110 + 1G > T at the first base downstream of the 3′-end of exon 30. One *NF1* gene mosaic variant was found in a proband who complained of cutaneous neurofibroma with the frameshift variant c.495_498del:p.Thr165fs in exon 5, and ultradeep sequencing showed the highest mutation rate of 10.81% in cutaneous neurofibromas. A frameshift variant, c.36_39del:p.Ser12fs in exon 1 of the *NF2* gene, was found in a proband who presented with skin plaques and intracranial neurogenic tumors. All of these pathogenic variants were heterozygous, one was not reported, and one not in Chinese before. This study expands the pathogenic variant spectrum of NF and demonstrates the clinical diagnosis.

## Introduction

Neurofibromatosis (NF) is characterized by abnormal development of the nervous system, bones, and skin. It can be divided into the following three different clinical types: NF type 1 (NF1), NF type 2 (NF2), and schwannomatosis. The most common form is NF1 (96%), followed by NF2 (3%) and the lesser known form schwannomatosis (Kresak and Walsh, 2016). The incidence rates of NF1, NF2, and schwannomatosis are 1:3,000, 1:60,000, and 1:70,000 in the general population, respectively (Dhamija et al., 1993; Evans, 1993; Friedman, 1993).

The clinical manifestations of NF are complex. Superficial features of NF1 include axillary, inguinal freckling, cafè-au-lait macules (CALMs), multiple cutaneous neurofibromas, and iris Lisch nodules. NF2 is characterized by bilateral vestibular schwannomas (VSs) with associated symptoms of hearing loss, tinnitus, and balance dysfunction (Evans, 1993). Schwannomatosis is prone to peripheral nerve sheath tumors. Histological features of cutaneous neurofibromas showed tumoral nodules with wavy spindle cell changes. The expression of the S100 protein in the cytoplasm and nucleus highlights Schwann cell elements, while CD34 showed a specialized fibroblastic component forming a net-like pattern and presenting NF architectures (Miettinen et al., 2017).

Neurofibromatosis is an autosomal-dominant disorder. Approximately 50% of individuals inherit it from a parent, while in others it is caused by a spontaneous mutation (Friedman, 1993). NF1 and NF2 are caused by mutations in *NF1* at 17q11.2 and *NF2* at 22q12, respectively. Mosaic NF type 1 (MNF1) is a somatic mosaicism of NF1 that is uncommon (Garcia-Romero et al., 2016). The clinical manifestations of MNF are similar to those of NF1. It is not easy to distinguish NF clinically because it has multiple and complicated phenotypes. Thus, molecular investigation is useful to identify pathogenic variants and improve diagnosis (Louvrier et al., 2018).

Next-generation sequencing (NGS) is a high-output sequencing method that can rapidly sequence exomes, transcriptomes, and genomes (Levy and Myers, 2016; Le Gallo et al., 2017). NGS, especially multigene panel testing, has promoted rapid progress, allowing for the simultaneous analysis of many genes and improvements in the detection rate of mutations (Shin et al., 2020). In this study, we applied multigene panel testing combined with Sanger sequencing testing to five families suspected of having NF and made clear diagnoses.

## Materials and Methods

### Study Subjects

Five probands suspected of having NF and nine relatives were from the outpatient department of the First Affiliated Hospital of Anhui Medical University. Clinical samples were collected, including peripheral blood, skin lesions, hair, oral mucosa, and cutaneous tissue. The study was approved by the Institutional Review Board of our hospital. Informed consent was obtained from all participants or their guardians for the collection of data and samples. The samples collected from the probands’ families are shown in Supplementary Table 1.

**FIGURE 1:**
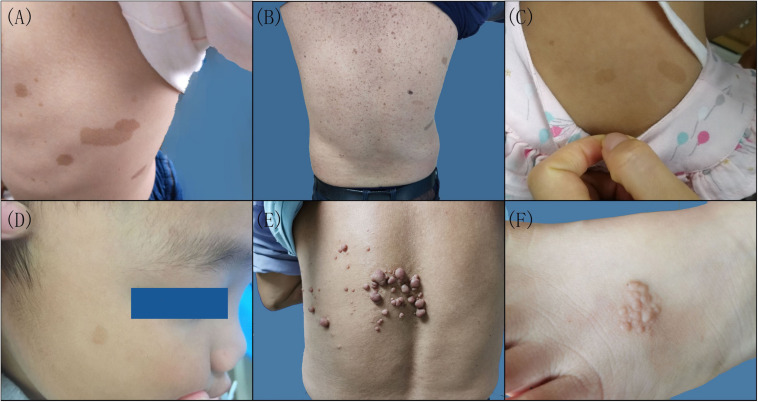
Clinical features of the patients with neurofibromatosis (NF). Multiple cafeÌ-au-lait macules were scattered on the left lateral chest of the proband **(A)** and the back of the proband’s father in family 1 **(B)**, the back of the proband in family 2 **(C)**, and the right side of the face of the proband in family 3 **(D)**. **(E)** Cutaneous neurofibroma on the back of the proband in family 4. **(F)** Skin pigmented plaques on the left foot back of the proband in family 5.

There were six patients in five families (Supplementary Figure 1). Three probands, including a 3-year-old girl (III-1) from family 1, a 2-year-old girl (II-1) from family 2, and a 2.8-year-old boy (II-1) from family 3, presented with scattered CALMs throughout the body (Figures 1A,C,D), and the father (III-1) of the proband from family 1 exhibited CALMs in the trunk area (Figure 1B). These patients were born with CALMs that gradually increased with age. These patients showed no other abnormalities. Reflectance confocal microscopy (RCM) (Supplementary Figure 2A) and dermoscopy (Supplementary Figure 2B) of the probands from family 2 and family 3 revealed significantly increased pigment contents in the stratum basal and regular sepia grid-like pigmentation with clear boundaries.

A 62-year-old man in family 4 (II-4) presented with cutaneous neurofibromas of the left back, chest, and abdomen at the age of 58 years (Figure 1E). No other abnormalities were found. Histological evaluation of the cutaneous neurofibromas showed a large and isolated tumoral nodule that presented wavy spindle cell changes (Supplementary Figures 3A,D). Immunohistochemical analysis showed scattered S100 positivity in tumor cell nuclei (Supplementary Figures 3C,F) and strong and diffuse CD34 positivity in the tumor cell cytoplasm (Supplementary Figures 3B,E). No abnormalities were found in the rest of the family members.

A 4-year-old female proband in family 5 (II-1) mainly presented with skin plaques, amblyopia in her right eye, and an intracranial neurogenic tumor by MRI. The skin plaque was present on her right foot when she was born and developed into six skin lesions as she grew up (Figure 1F). These lesions were distributed in the trunk area, left hip, right calf front knee, right ankle, and left foot back and consisted of well-circumscribed and slightly pigmented plaques. There were no obvious differences in learning ability and intelligence between her and her peers during her growth. Cranial MRI examination showed an intracranial neurogenic tumor located in the cerebellum medulla oblongata pool of the right side. Histological evaluation of the skin plaques showed multiple nodules forming well-circumscribed interconnected masses of different sizes (Supplementary Figure 4A). These multiple nodules presented swirly spindle cell changes (Supplementary Figure 4D). Immunohistochemical analysis showed strong and diffuse S100 positivity in the tumor cell nuclei (Supplementary Figures 4C,F) and weak and scattered CD34 positivity in the tumor cell cytoplasm (Supplementary Figures 4B,E). Other family members did not show similar symptoms.

All clinical manifestations in the NF families are shown in Table 1.

**TABLE 1:** Clinical characteristics of the neurofibromatosis proband study.

Family no.	Patient no.	Age (years)	Gender	CALMs	Neurofibromas	Intracranial neurogenic tumor	Lisch nodules	Ocular abnormalities
1	III-1	3	F	+	−	−	−	−
2	II-1	2	F	+	−	−	−	−
3	II-1	2.8	M	+	−	−	−	−
4	II-4	62	M	−	+	−	NA	−
5	II-1	4	F	+	+	+	NA	+

*“+”: affected; “−”: unaffected. *CALMs*, cafeì-au-lait macules; *NA*, not available.*

### DNA Isolation of Samples

Genomic DNA was extracted from the peripheral blood, skin lesions, hair, oral mucosa, and tumor tissues separately using the AxyPrep Blood Genomic DNA Miniprep Kit (Axygen, Corning, Jiangsu, China) and DNeasy Blood & Tissue Kit (Qiagen, Germany). The DNA purity was determined using a NanoDrop one spectrophotometer and quantified with a Qubit 2.0 Fluorometer using the Qubit dsDNA HS Assay Kit (Life Technologies, Thermo Fisher Scientific, Inc.) according to the manufacturer’s recommendations. DNA samples were then preserved at −20°C for further experiments.

### Multigene Panel Testing

To explore the genetic properties of the patients, the capture probe from the Roche NimbleGen Sequence Capture SeqCap EZ Library was used to capture a total of 569 genes associated with hereditary dermatosis. Firstly, DNA was cropped into approximately 300-bp fragments using focused ultrasonicators (Covaris M220, United States) and used to construct the DNA library. Then, streptavidin-coated magnetic beads by NimbleGen (Roche NimbleGen, Inc.) were bound to an avidin-labeled probe after the probe had captured the target exons. Next, a hybridization reaction between the DNA library with various index marks and probes with biotin was performed. After linear PCR amplification, the quality of the library was determined. Sequencing was carried out on an Illumina HiSeq X Ten System (Illumina, San Diego, CA, United States) under sequencing efficiency with an average sequencing depth > 200× and Q30 > 90% according to the manufacturer’s instructions. DNA from all of the probands’ blood samples and tumor tissues from the proband in family 4 were subjected to multigene panel testing.

### Sanger Sequencing

Sanger sequencing was used to confirm the mutations found by multigene panel testing, which were analyzed to determine whether the variants co-segregated with the disease phenotype in their families. Primers were designed based on the mutation sites found using Primer 3.0. An ABI PRISM 3730XL analyzer (Applied Biosystems, Foster City, CA, United States) was used for Sanger sequencing. The DNA from blood samples of all the probands and their relatives was assessed separately, and the mutation sites of the genes were detected by multigene panel testing.

### Ultradeep Sequencing

When multigene panel testing could detect mutations in the probands’ tumor tissues but not in the peripheral blood, ultradeep sequencing was applied to determine the mutation percentage in the peripheral blood, skin lesions, hair, oral mucosa, and tumor tissues. This was performed using the Illumina HiSeq X Ten System (Illumina, San Diego, CA, United States) after DNA quantification and library quality control. The variant allele frequency (VAF) was defined as the number of reads that mapped to whole exons of causal genes, including untranslated and splicing regions. A mutation was considered present when the mutation site VAF was >1.0%.

## Results

Four *NF1* variants in four families and one *NF2* variant in one family were found in our study. All variants were heterozygous, and the genetic findings and analyses are shown in Table 2.

**TABLE 2:** Variants identified of neurofibromatosis in this study.

Nucleotide change	Mutation gene	Amino acid change	Variant type	Type of variants	Reference	Inheritance	SIFT_score	Mutation Taster_score
c.5072_5073insTATAACTGTAACT CCTGGGTCAGGGAGTACACCAA	*NF1*	p.Tyr1692Ilefs	Frameshift	Germline	Novel	Familial	0	0
c.4110 + 1G > T	*NF1*	NA	Splicing	Germline	Reported	Sporadic	0	1
c.3826C > T	*NF1*	p.Arg1276Ter	Missense	Germline	Reported	Sporadic	1	1
c.495_498del	*NF1*	p.Thr165fs	Frameshift	Mosaicism	Reported	Sporadic	0	0
c.36_39del	*NF2*	p.Ser12fs	Frameshift	Germline	Reported	Sporadic	0	0

**NA*, not available.*

**FIGURE 2:**
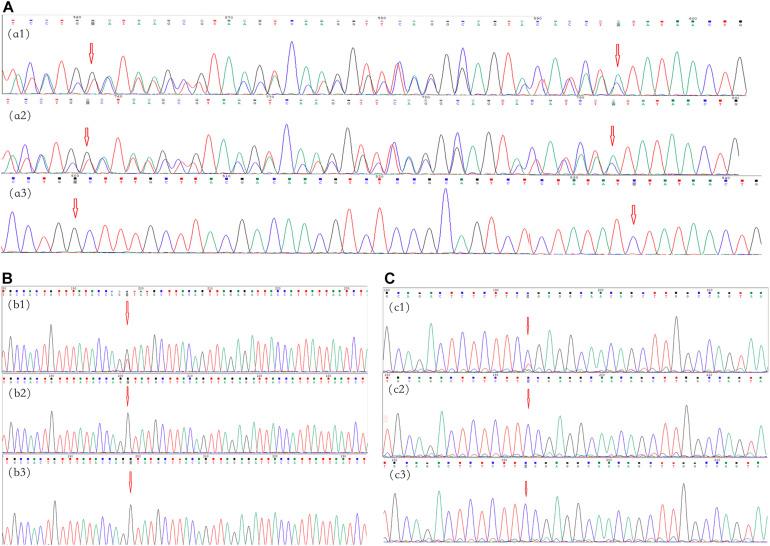
Mutational analysis of the neurofibromatosis (NF) patients in families 1–3. The *red arrows* indicate the mutation sites. **(A)** Family 1: the c.5072_5073insTATAACTGTAACTCCTGGGTCAGGGAGTACACCAA:p.Tyr1692Ilefs frameshift variant in *NF1* in exon 37 in proband III-1 **(*a1*)** and her father **(*a2*)**; there was no mutation in her mother **(*a3*)**. **(B)** Family 2: the c.4110 + 1G > T splicing variant in NF1 at the first base downstream of the 3′-end of exon 30 in proband II-1 **(b1)**; there were no mutations in her father and mother (**b2,b3**, respectively). **(C)** Family 3: the c.3826C > T:p.Arg1276Ter missense variant in NF1 in exon 28 in proband II-1 **(c1)**; there were no mutations in his father and mother (**c1,c2**, respectively).

A novel frameshift variant, c.5072_5073insTATAACTGTAA CTCCTGGGTCAGGGAGTACACCAA:p.Tyr1692Ilefs in exon 37, was found in III-1 and II-3 in family 1 (Figure 2A), which inserted 12 amino acids and changed the amino acid sequence starting at position 1692, followed by the production of abnormal neurofibromin. A splicing variant, c.4110 + 1G > T, at the first base downstream of the 3′-end of exon 30 was found in II-1 in family 2 (Figure 2B), which resulted in putative aberrant splicing. A missense variant, c.3826C > T:p.Arg1276Ter in exon 28, was identified in II-1 in family 3 (Figure 2C), which resulted in the replacement of the arginine residue at 1276 position with a stop codon and premature protein truncation. These variants were not found in the DNA samples from unaffected family members.

**FIGURE 3:**
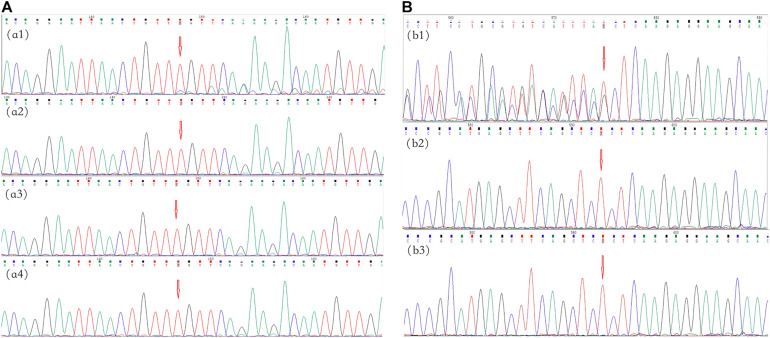
Mutational analysis of the neurofibromatosis (NF) patients in families 4 and 5. The *red arrows* indicate the mutation sites. **(A)** Family 4: the c.495_498del:p.Thr165fs frameshift variant in *NF1* in exon 5 in cutaneous neurofibromas from proband II-4 **(*a1*)**; there was no mutation in the peripheral blood, oral mucosa, or hair (***a2*–*a4***, respectively). **(B)** Family 5: the c.36_39del:p.Ser12fs frameshift variant in *NF2* in exon 1 in proband II-1 **(*b1*)**; there were no mutations in her father and mother (***b2*,*b3***, respectively).

In family 4, no variant was found in the peripheral blood of the proband (II-4) by multigene panel testing. However, testing of cutaneous neurofibromas from the proband showed a frameshift variant, c.495_498del:p.Thr165fs, in exon 5 of *NF1* (Figure 3A). The pathogenic variant deleted four bases, including thymine, guanine, thymine, and thymine, located from 495–498 in *NF1* exon 5, which caused a change in the amino acid at position 165. This result was confirmed in the cutaneous neurofibroma tissue of the proband by Sanger sequencing. Ultradeep sequencing analysis was used to determine the mutation percentage of samples, including from the peripheral blood, the oral mucosa, hair follicles, and cutaneous neurofibromas. The highest mutation rate of 10.81% was observed in the cutaneous neurofibromas, while those of other tissues were less than 0.01% (Supplementary Table 2). These results confirmed that the proband had MNF1. This variant was not found in the DNA derived from the peripheral blood of his unaffected daughter.

In family 5, the genetic testing analysis presented a frameshift variant, c.36_39del:p.Ser12fs in exon 1 of *NF2*, in the proband’s peripheral blood (Figure 3B). The absence of bases 36–39 in exon 1 resulted in the deletion of the serine residue at position 12. The variant was not found in the unaffected family members.

These variants were also not found in the DNA samples from 100 unrelated healthy volunteers.

## Discussion

The main manifestation of CALMs can overlap with that of other disorders, such as Legius syndrome (Brems et al., 2012), Lynch syndrome (Wimmer et al., 2017), and the Piebald trait (Stevens et al., 2012). According to some studies, the incidence rates of *NF1* and *SPRED1* mutations in NF1 patients presenting with familial CALMs are 73 and 19%, respectively (Messiaen and Legius, 2010). In this study, three probands only presented with CALMs. RCM and dermoscopy could provide new ways to help diagnose CALMs, which were used as supplementary diagnostic criteria for NF1 (Duman and Elmas, 2015). The results of the RCM and dermoscopy examination showed pigmentation changes, supporting the observed CALMs.

*NF1* is a highly mutable tumor suppressor gene, and approximately half of affected individuals have *de novo* variants (Yao et al., 2019). Approximately half of children with NF1 have no known family history (Friedman, 1993). Genetic testing could confirm the diagnosis of NF1 in patients suspected of NF1 but only presenting with CALMs (Wang et al., 2019). The variant c.5072_5073insTATAACTGTAACTCCTGGGTCAGGGAGTAC ACCAA:p.Tyr1692Ilefs in exon 37 was found both in the proband and her father from family 1, so the mutation was inherited from her father. This variant was not reported before.

In family 2, the splicing variant, c.4110 + 1G > T of *NF1* at the first base downstream of the 3′-end of exon 30, affects a donor splice site in intron 30 of the *NF1* gene, which is expected to disrupt RNA splicing and likely result in an absent or disrupted protein product. This mutation was reported in NF1 patients of German or Turkish descent (Fahsold et al., 2000). It was first found in the Chinese NF1 patient in this study. The variant c.3826C > T:p.Arg1276Ter in exon 28 was found in the proband from family 3. This was first reported in two unrelated patients from Spain (Valero et al., 2011) and identified in Chinese and other populations later (Fahsold et al., 2000; Zhang et al., 2015). The sporadic NF1 variant was most likely a *de novo* variant or inherited from the germ cell of one or both of the parents (Friedman, 1993). No genetic testing was performed on the germ cells from the parents of the probands (families 2 and 3), so the source of the mutation is unclear.

MNF1 is an uncommon NF1 subtype with a prevalence rate of approximately 0.0006–0.0027% (Wolkenstein et al., 1995; Ruggieri and Huson, 2001; Ruggieri et al., 2004; Pascual-Castroviejo et al., 2008; Cohen, 2016). In general, affected individuals exhibit milder phenotypes than complete NF1. Fernandez-Rodriguez et al. (2011) revealed the possible biological mechanisms, which were somatic mosaicism and the presence of mild NF1 mutations. Cutaneous neurofibromas are the most common clinical manifestation, with a prevalence of 56% in MNF1 (Ruggieri and Huson, 2001; Wagner et al., 2018). The proband in family 4 only presented with large cutaneous neurofibromas in some parts of the body. The histopathology results showed wavy spindle cell changes, and immunohistochemical analysis mainly presented strong and diffuse CD34 positivity, which supported the neurofibroma changes.

No variant was found in the peripheral blood of the proband in family 4, but a frameshift variant, c.495_498del:p.Thr165fs in exon 5 of *NF1*, was discovered in his cutaneous neurofibromas, which may produce an abnormal protein product. Ultradeep sequencing showed that the frequency of the mutant *NF1* gene was the highest at 10.81% in cutaneous neurofibromas, though it was very low in the peripheral blood, hair, or the oral mucosa from the patient or normal control. This patient was found to be a somatic mosaic. All three germ layers should carry mutations, and the clinical phenotype will be generalized disease if the mutation occurs earlier than zygote formation. Mutations occurring later in development during embryogenesis may affect fewer cell types or specific tissues. The proband’s children might have a risk of classic NF1 if the affected individuals display gonosomal mosaicism (Hardin et al., 2014; Garcia-Romero et al., 2016). However, there was no molecular proof revealing the proband to be an NF1 gonosomal mosaic because the proband refused to provide his semen. Currently, no clinical manifestation of NF has appeared in his offsprings, including a son and two daughters. The variant might be a *de novo* mutation and occurred in the late stage of embryo development after zygote formation. This mutation has been reported in the germline (Osborn and Upadhyaya, 1999; Toliat et al., 2000; Ars et al., 2003; Schaefer et al., 2013; Xu et al., 2014) and saliva (Toliat et al., 2000) from multiple patients with sporadic or familial NF1, which belong to the germline mutation. Interestingly, it is the first report of c.499_502delTGTT appearing as the somatic mosaicism variant.

NF2 is a devastating autosomal-dominant disorder and is characterized by VS (Evans, 1993; Kluwe and Mautner, 1998). The early and typical features of NF2 are meningiomas, ocular abnormalities, or skin plaques in children (Evans et al., 1999; Ruggieri et al., 2015; Gaudioso et al., 2019). The diagnosis of NF2 is easy in adults, but it is often delayed in pediatric patients. The average age of these diagnoses in individuals is 18–24 years, or even earlier (Evans, 1993). The prognosis of younger individuals affected with NF2 might be worse, and severe sporadic NF2 typically occurs in childhood. Skin plaques were the initial manifestation in the proband in family 5. Histological and immunohistochemical analyses showed a dermal plexiform schwannoma with swill spindle cell changes and a strong and diffuse S100 staining. Furthermore, amblyopia was found in her right eye, and an approximately 12-mm tumor was located in the cerebellum medulla oblongata pool of the right brain. Intracranial tumors might become larger, form multiple neurogenic tumors, and further press the tongue base, which could cause epilepsy and affect her ability to speak.

A heterozygous pathogenic frameshift variant, c.36_39del:p.Ser12fs in exon 1 of *NF2*, was found in the proband of family 5, which might result in abnormally truncated proteins because the reading frameshift was predicted to introduce a premature stop codon. It was reported as the somatic inactivating mutation in a study for detecting *SMARCB1* and *NF2* gene mutations (Paganini et al., 2018). It is, for the first time, reported as a germline mutation in this report, which suggests that the same mutation may occur at the different stages of embryo formation.

Approximately 50% of patients with NF2 have an affected parent, while the remaining 50% may have a *de novo* mutation (Mills et al., 2018). Approximately 25–33% of patients with a *de novo NF2* variant have somatic mosaicism (Kluwe et al., 2003; Moyhuddin et al., 2003; Evans et al., 2007, 2013). Genetic testing showed an *NF2* variant in her blood lymphocytes, but no mutation in her parents. However, we did not examine non-hematopoietic tissues from the proband or her parents, specifically germ cells. The presentation may be due to germline mosaicism in a parent or a *de novo* pathogenic variant in the proband. According to genotype–phenotype correlations, frameshift mutations are frequently associated with meningioma (Baser et al., 2004; Smith et al., 2011). Therefore, the prognosis may not be optimistic for the 4-year-old girl.

Multigene panel belongs to next-generation sequencing, and multiple genes can be tested at one time. It has a higher gene mutation detection rate. By applying multigene panel testing methods, the molecular cause has been successfully elucidated in 91% of 35 inherited ichthyosis patients (Cheng et al., 2020) and 90% of 40 suspected epidermolysis bullosa patients (Has et al., 2018). Although it is not a 100% discovery rate, there is undoubtedly an economic and a time advantage to panel genetic testing *versus* a stepwise approach, which is one of the alternative effective methods to help clinical diagnosis. In this study, five affected probands with suspected NF from unrelated families were enrolled. The probands had different clinical manifestations (CALMs, cutaneous neurofibromas, skin plaque, and intracranial neurogenic tumor), and it was difficult to diagnose and determine the type of NF based on clinical manifestations alone. Fortunately, all of them were diagnosed clearly using multigene panel testing combined with Sanger sequencing.

Multigene panel testing combined with Sanger sequencing was used to test five probands suspected of having NF. Three *NF1* mutations were found in three probands with CALMs, one *NF1* mosaic variant was observed in a proband with cutaneous neurofibroma, and an *NF2* variant was found in a proband with skin plaques and an intracranial neurogenic tumor. All of these variants were present in the heterozygous state; one of them has never been reported previously, and one has been reported in the Chinese population for the first time. The additional methods employed in the present study, including multigene panel testing, Sanger sequencing, and ultradeep sequencing, helped to clarify the diagnosis. The novel pathogenic variants expanded the pathogenic variant spectrum of NF.

## Data Availability Statement

The datasets for this article are not publicly available due to concerns regarding participant/patient anonymity. Requests to access the datasets should be directed to the corresponding author.

## Ethics Statement

The studies involving human participants were reviewed and approved by Institutional Review Board of the First Affiliated Hospital of Anhui Medical University. Written informed consent to participate in this study was provided by the participants’ legal guardian/next of kin. Written informed consent was obtained from the minor(s)’ legal guardian/next of kin for the publication of any potentially identifiable images or data included in this article.

## Author Contributions

F-LX and W-LF conceived and designed the study and revised the manuscript. Z-Y-OZ and Y-YW wrote the manuscript. XC helped perform the experiments and prepared the samples in this study. All the authors reviewed the manuscript.

## Conflict of Interest

The authors declare that the research was conducted in the absence of any commercial or financial relationships that could be construed as a potential conflict of interest.

## Supplementary Material

Supplementary Figure 1 Family pedigree of the five probands with NF. “⏹”: affected male individual. “⏺”: affected female individual. “◻”: unaffected male individual. “⭘”: unaffected female individual. “↗”: probands in the family “” dead male individual in the family.

Supplementary Figure 2 Pictures of CALMs by reflectance confocal microscopy and dermoscopy examination. High refractive index particles of different sizes in the superficial dermis (red arrow) significantly increased pigment contents in the stratum layer (yellow arrows) under RCM **(A)**. Regular sepia grid-like pigmentation under a microscope with clear boundaries by dermoscopy (100X) **(B)**.

Supplementary Figure 3 Histological and immunohistochemical manifestations of cutaneous neurofibromas in the proband from family 4. A large and isolated nodule in the dermis **(A**, 10X) and wavy spindle cell changes (**D**, 40X). Strong and diffuse CD34 staining (**B**, 10X) and strong CD34 staining in the tumor cell cytoplasm (**E**, 40X). Scattered S100 staining (**C**, 10X) and S100 staining in tumor cell nuclei (**F**, 40X).

Supplementary Figure 4 Histological and immunohistochemical manifestations of skin plaques in the proband from family 5. The nodules interconnected with each other (**A**, 10X) and the change in swirly spindle cells (**D**, 40X). Scattered CD34 staining (**B**, 10X) and weak CD34 staining in the tumor cell cytoplasm (**E**, 40X). Strong and diffuse S100 staining (**C**, 10X) and strong S100 staining in the tumor cell nuclei (**F**, 40X).

## References

[B1] Ars E. Kruyer H. Morell M. Pros E. Serra E. Ravella A. (2003). Recurrent mutations in the NF1 gene are common among neurofibromatosis type 1 patients. *J. Med. Genet.* 40:e82. 10.1136/jmg.40.6.e82 12807981 PMC1735494

[B2] Baser M. E. Kuramoto L. Joe H. Friedman J. M. Wallace A. J. Gillespie J. E. (2004). Genotype-phenotype correlations for nervous system tumors in neurofibromatosis 2: a population-based study. *Am. J. Hum. Genet.* 75 231–239. 10.1086/422700 15190457 PMC1216057

[B3] Brems H. Pasmant E. Van Minkelen R. Wimmer K. Upadhyaya M. Legius E. (2012). Review and update of SPRED1 mutations causing Legius syndrome. *Hum. Mutat.* 33 1538–1546. 10.1002/humu.22152 22753041

[B4] Cheng R. Liang J. Li Y. Zhang J. Ni C. Yu H. (2020). Next-generation sequencing through multi-gene panel testing for diagnosis of hereditary ichthyosis in Chinese. *Clin. Genet.* 97 770–778. 10.1111/cge.13704 31953843

[B5] Cohen P. R. (2016). Segmental neurofibromatosis and cancer: report of triple malignancy in a woman with mosaic Neurofibromatosis 1 and review of neoplasms in segmental neurofibromatosis. *Dermatol. Online J.* 22:13030/qt66k5j4wt. 27617721

[B6] Dhamija R. Plotkin S. Asthagiri A. Messiaen L. Babovic-Vuksanovic D. (1993). “Schwannomatosis,” in *GeneReviews((R))*, eds Adam M. P. Ardinger H. H. Pagon R. A. Wallace S. E. Bean L. J. H. Stephens K. (Seattle, WA: University of Washington).

[B7] Duman N. Elmas M. (2015). Dermoscopy of cutaneous neurofibromas associated with neurofibromatosis type 1. *J. Am. Acad. Dermatol.* 73 529–531. 10.1016/j.jaad.2015.05.021 26282805

[B8] Evans D. G. (1993). “Neurofibromatosis 2,” in *GeneReviews((R))*, eds Adam M. P. Ardinger H. H. Pagon R. A. Wallace S. E. Bean L. J. H. Stephens K. (Seattle, WA: University of Washington).

[B9] Evans D. G. Birch J. M. Ramsden R. T. (1999). Paediatric presentation of type 2 neurofibromatosis. *Arch. Dis. Child* 81 496–499. 10.1136/adc.81.6.496 10569966 PMC1718148

[B10] Evans D. G. Bowers N. Huson S. M. Wallace A. (2013). Mutation type and position varies between mosaic and inherited NF2 and correlates with disease severity. *Clin. Genet.* 83 594–595. 10.1111/cge.12007 22989157

[B11] Evans D. G. Ramsden R. T. Shenton A. Gokhale C. Bowers N. L. Huson S. M. (2007). Mosaicism in neurofibromatosis type 2: an update of risk based on uni/bilaterality of vestibular schwannoma at presentation and sensitive mutation analysis including multiple ligation-dependent probe amplification. *J. Med. Genet.* 44 424–428. 10.1136/jmg.2006.047753 17307835 PMC2598002

[B12] Fahsold R. Hoffmeyer S. Mischung C. Gille C. Ehlers C. Kucukceylan N. (2000). Minor lesion mutational spectrum of the entire NF1 gene does not explain its high mutability but points to a functional domain upstream of the GAP-related domain. *Am. J. Hum. Genet.* 66 790–818. 10.1086/302809 10712197 PMC1288164

[B13] Fernandez-Rodriguez J. Castellsague J. Benito L. Benavente Y. Capella G. Blanco I. (2011). A mild neurofibromatosis type 1 phenotype produced by the combination of the benign nature of a leaky NF1-splice mutation and the presence of a complex mosaicism. *Hum. Mutat.* 32 705–709. 10.1002/humu.21500 21394830

[B14] Friedman J. M. (1993). “Neurofibromatosis 1,” in *GeneReviews((R))*, eds Adam M. P. Ardinger H. H. Pagon R. A. Wallace S. E. Bean L. J. H. Stephens K. (Seattle, WA: University of Washington). 20301288

[B15] Garcia-Romero M. T. Parkin P. Lara-Corrales I. (2016). Mosaic neurofibromatosis type 1: a systematic review. *Pediatr. Dermatol.* 33 9–17. 10.1111/pde.12673 26338194

[B16] Gaudioso C. Listernick R. Fisher M. J. Campen C. J. Paz A. Gutmann D. H. (2019). Neurofibromatosis 2 in children presenting during the first decade of life. *Neurology* 93 e964–e967. 10.1212/WNL.0000000000008065 31363058

[B17] Hardin J. Behm A. Haber R. M. (2014). Mosaic generalized neurofibromatosis 1: report of two cases. *J. Cutan. Med. Surg.* 18 271–274. 10.2310/7750.2013.13116 25008444

[B18] Has C. Kusel J. Reimer A. Hoffmann J. Schauer F. Zimmer A. (2018). The position of targeted next-generation sequencing in epidermolysis bullosa diagnosis. *Acta Derm. Venereol.* 98 437–440. 10.2340/00015555-2863 29242947

[B19] Kluwe L. Mautner V. Heinrich B. Dezube R. Jacoby L. B. Friedrich R. E. (2003). Molecular study of frequency of mosaicism in neurofibromatosis 2 patients with bilateral vestibular schwannomas. *J. Med. Genet.* 40 109–114. 10.1136/jmg.40.2.109 12566519 PMC1735360

[B20] Kluwe L. Mautner V. F. (1998). Mosaicism in sporadic neurofibromatosis 2 patients. *Hum. Mol. Genet.* 7 2051–2055. 10.1093/hmg/7.13.2051 9817921

[B21] Kresak J. L. Walsh M. (2016). Neurofibromatosis: a review of NF1. NF2, and Schwannomatosis. *J. Pediatr. Genet.* 5 98–104. 10.1055/s-0036-1579766 27617150 PMC4918700

[B22] Le Gallo M. Lozy F. Bell D. W. (2017). Next-generation sequencing. *Adv. Exp. Med. Biol.* 943 119–148. 10.1007/978-3-319-43139-0_5 27910067

[B23] Levy S. E. Myers R. M. (2016). Advancements in next-generation sequencing. *Annu. Rev. Genomics Hum. Genet.* 17 95–115. 10.1146/annurev-genom-083115-022413 27362342

[B24] Louvrier C. Pasmant E. Briand-Suleau A. Cohen J. Nitschke P. Nectoux J. (2018). Targeted next-generation sequencing for differential diagnosis of neurofibromatosis type 2, schwannomatosis, and meningiomatosis. *Neuro Oncol.* 20 917–929. 10.1093/neuonc/noy009 29409008 PMC6007397

[B25] Messiaen L. Legius E. (2010). Error in a study of the clinical and mutational spectrum of neurofibromatosis type 1-like syndrome. *JAMA* 303 2476–2477. 10.1001/jama.2010.827 20571013

[B26] Miettinen M. M. Antonescu C. R. Fletcher C. D. M. Kim A. Lazar A. J. Quezado M. M. (2017). Histopathologic evaluation of atypical neurofibromatous tumors and their transformation into malignant peripheral nerve sheath tumor in patients with neurofibromatosis 1-a consensus overview. *Hum. Pathol.* 67 1–10. 10.1016/j.humpath.2017.05.010 28551330 PMC5628119

[B27] Mills J. R. Moyer A. M. Kipp B. R. Poplawski A. B. Messiaen L. M. Babovic-Vuksanovic D. (2018). Unilateral vestibular schwannoma and meningiomas in a patient with PIK3CA-related segmental overgrowth: co-occurrence of mosaicism for 2 rare disorders. *Clin. Genet.* 93 187–190. 10.1111/cge.13099 28737257

[B28] Moyhuddin A. Baser M. E. Watson C. Purcell S. Ramsden R. T. Heiberg A. (2003). Somatic mosaicism in neurofibromatosis 2: prevalence and risk of disease transmission to offspring. *J. Med. Genet.* 40 459–463. 10.1136/jmg.40.6.459 12807969 PMC1735486

[B29] Osborn M. J. Upadhyaya M. (1999). Evaluation of the protein truncation test and mutation detection in the NF1 gene: mutational analysis of 15 known and 40 unknown mutations. *Hum. Genet.* 105 327–332. 10.1007/s004399900135 10543400

[B30] Paganini I. Capone G. L. Vitte J. Sestini R. Putignano A. L. Giovannini M. (2018). Double somatic SMARCB1 and NF2 mutations in sporadic spinal schwannoma. *J. Neurooncol.* 137 33–38. 10.1007/s11060-017-2711-6 29230670

[B31] Pascual-Castroviejo I. Pascual-Pascual S. I. Viano J. (2008). Segmental neurofibromatosis type 1 (NF1) associated with Cobb syndrome: case report. *Neuropediatrics* 39 341–343. 10.1055/s-0029-1214422 19568998

[B32] Ruggieri M. Huson S. M. (2001). The clinical and diagnostic implications of mosaicism in the neurofibromatoses. *Neurology* 56 1433–1443. 10.1212/wnl.56.11.1433 11409413

[B33] Ruggieri M. Pavone P. Polizzi A. Di Pietro M. Scuderi A. Gabriele A. (2004). Ophthalmological manifestations in segmental neurofibromatosis type 1. *Br. J. Ophthalmol.* 88 1429–1433. 10.1136/bjo.2004.043802 15489488 PMC1772378

[B34] Ruggieri M. Pratico A. D. Evans D. G. (2015). Diagnosis, management, and new therapeutic options in childhood neurofibromatosis Type 2 and related forms. *Semin. Pediatr. Neurol.* 22 240–258. 10.1016/j.spen.2015.10.008 26706012

[B35] Schaefer I. M. Strobel P. Thiha A. Sohns J. M. Muhlfeld C. Kuffer S. (2013). Soft tissue perineurioma and other unusual tumors in a patient with neurofibromatosis type 1. *Int. J. Clin. Exp. Pathol.* 6 3003–3008. 24294391 PMC3843285

[B36] Shin H. C. Lee H. B. Yoo T. K. Lee E. S. Kim R. N. Park B. (2020). Detection of germline mutations in breast cancer patients with clinical features of hereditary cancer syndrome using a multi-gene panel test. *Cancer Res. Treat.* 52 697–713. 10.4143/crt.2019.559 32019277 PMC7373875

[B37] Smith M. J. Higgs J. E. Bowers N. L. Halliday D. Paterson J. Gillespie J. (2011). Cranial meningiomas in 411 neurofibromatosis type 2 (NF2) patients with proven gene mutations: clear positional effect of mutations, but absence of female severity effect on age at onset. *J. Med. Genet.* 48 261–265. 10.1136/jmg.2010.085241 21278391

[B38] Stevens C. A. Chiang P. W. Messiaen L. M. (2012). Cafe-au-lait macules and intertriginous freckling in piebaldism: clinical overlap with neurofibromatosis type 1 and Legius syndrome. *Am. J. Med. Genet. A* 158A 1195–1199. 10.1002/ajmg.a.35297 22438235

[B39] Toliat M. R. Erdogan F. Gewies A. Fahsold R. Buske A. Tinschert S. (2000). Analysis of the NF1 gene by temperature gradient gel electrophoresis reveals a high incidence of mutations in exon 4b. *Electrophoresis* 21 541–544. 10.1002/(SICI)1522-2683(20000201)21:3<541::AID-ELPS541<3.0.CO;2-L 10726756

[B40] Valero M. C. Martin Y. Hernandez-Imaz E. Marina Hernandez A. Melean G. Valero A. M. (2011). A highly sensitive genetic protocol to detect NF1 mutations. *J. Mol. Diagn.* 13 113–122. 10.1016/j.jmoldx.2010.09.002 21354044 PMC3128626

[B41] Wagner G. Meyer V. Sachse M. M. (2018). [Segmental neurofibromatosis]. *Hautarzt* 69 487–490. 10.1007/s00105-017-4078-1 29119198

[B42] Wang W. Qin W. Ge H. Kong X. Xie C. Tang Y. (2019). Clinical and molecular characteristics of thirty NF1 variants in Chinese patients with neurofibromatosis type 1. *Mol. Biol. Rep.* 46 4349–4359. 10.1007/s11033-019-04888-3 31201679

[B43] Wimmer K. Rosenbaum T. Messiaen L. (2017). Connections between constitutional mismatch repair deficiency syndrome and neurofibromatosis type 1. *Clin. Genet.* 91 507–519. 10.1111/cge.12904 27779754

[B44] Wolkenstein P. Mahmoudi A. Zeller J. Revuz J. (1995). More on the frequency of segmental neurofibromatosis. *Arch. Dermatol.* 131:1465. 10.1001/archderm.1995.01690240131030 7492147

[B45] Xu W. Yang X. Hu X. Li S. (2014). Fifty-four novel mutations in the NF1 gene and integrated analyses of the mutations that modulate splicing. *Int. J. Mol. Med.* 34 53–60. 10.3892/ijmm.2014.1756 24789688 PMC4072343

[B46] Yao R. Yu T. Xu Y. Yu L. Wang J. Wang X. (2019). Clinical presentation and novel pathogenic variants among 68 Chinese Neurofibromatosis 1 children. *Genes* 10:847. 10.3390/genes10110847 31717729 PMC6896037

[B47] Zhang J. Tong H. Fu X. Zhang Y. Liu J. Cheng R. (2015). Molecular characterization of NF1 and Neurofibromatosis Type 1 genotype-phenotype correlations in a Chinese population. *Sci. Rep.* 5:11291. 10.1038/srep11291 26056819 PMC4460887

