# Supplementary material for: Molecular Diagnosis of Neurofibromatosis by Multigene Panel Testing
Source: Front Genet. 2021 Mar 9;12:603195. doi: 10.3389/fgene.2021.603195 (PMC7985060; doi:10.3389/fgene.2021.603195)
Supplement: Supplementary file 1 [file Table_1.DOCX]

Supplementary Table 1 The clinical samples study of neurofibromatosis families

| Family No. | Patient No. | Peripheral blood | Hair | Oral mucosa | Cutaneous tissue |
| --- | --- | --- | --- | --- | --- |
| 1 | III-1 | + | - | - | - |
| 1 | II-3 | + | - | - | - |
| 1 | II-4 | + | - | - | - |
| 2 | II-1 | + | - | - | - |
| 2 | I-1 | + | - | - | - |
| 2 | I-2 | + | - | - | - |
| 3 | II-1 | + | - | - | - |
| 3 | I-1 | + | - | - | - |
| 3 | I-2 | + | - | - | - |
| 4 | II-4 | + | + | + | + |
| 4 | III-1 | + | - | - | - |
| 5 | II-1 | + | - | - | - |
| 5 | I-1 | + | - | - | - |
| 5 | I-2 | + | - | - | - |

Abbreviations: “+”: samples collected; “-”: sample unaffected
