# Supplementary material for: Molecular Diagnosis of Neurofibromatosis by Multigene Panel Testing
Source: Front Genet. 2021 Mar 9;12:603195. doi: 10.3389/fgene.2021.603195 (PMC7985060; doi:10.3389/fgene.2021.603195)
Supplement: Supplementary file 2 [file Table_2.DOCX]

Supplementary Table 2 The results of ultra-deep sequencing analysis for the proband of family 4

| Sample | Ref | Alt | Total | Percentage of alt |
| --- | --- | --- | --- | --- |
| Negative control | 62554 | 1 | 62555 | 0.0016% |
| Blood | 55326 | 5 | 55331 | 0.0090% |
| Oral mucosal | 39754 | 4 | 39758 | 0.0101% |
| Hair | 37193 | 1 | 37194 | 0.0027% |
| Neurofibromas | 45110 | 5465 | 50575 | 10.8057% |
