# Supplementary figures and images for: Molecular Diagnosis of Neurofibromatosis by Multigene Panel Testing
Source: Front Genet. 2021 Mar 9;12:603195. doi: 10.3389/fgene.2021.603195 (PMC7985060; doi:10.3389/fgene.2021.603195)

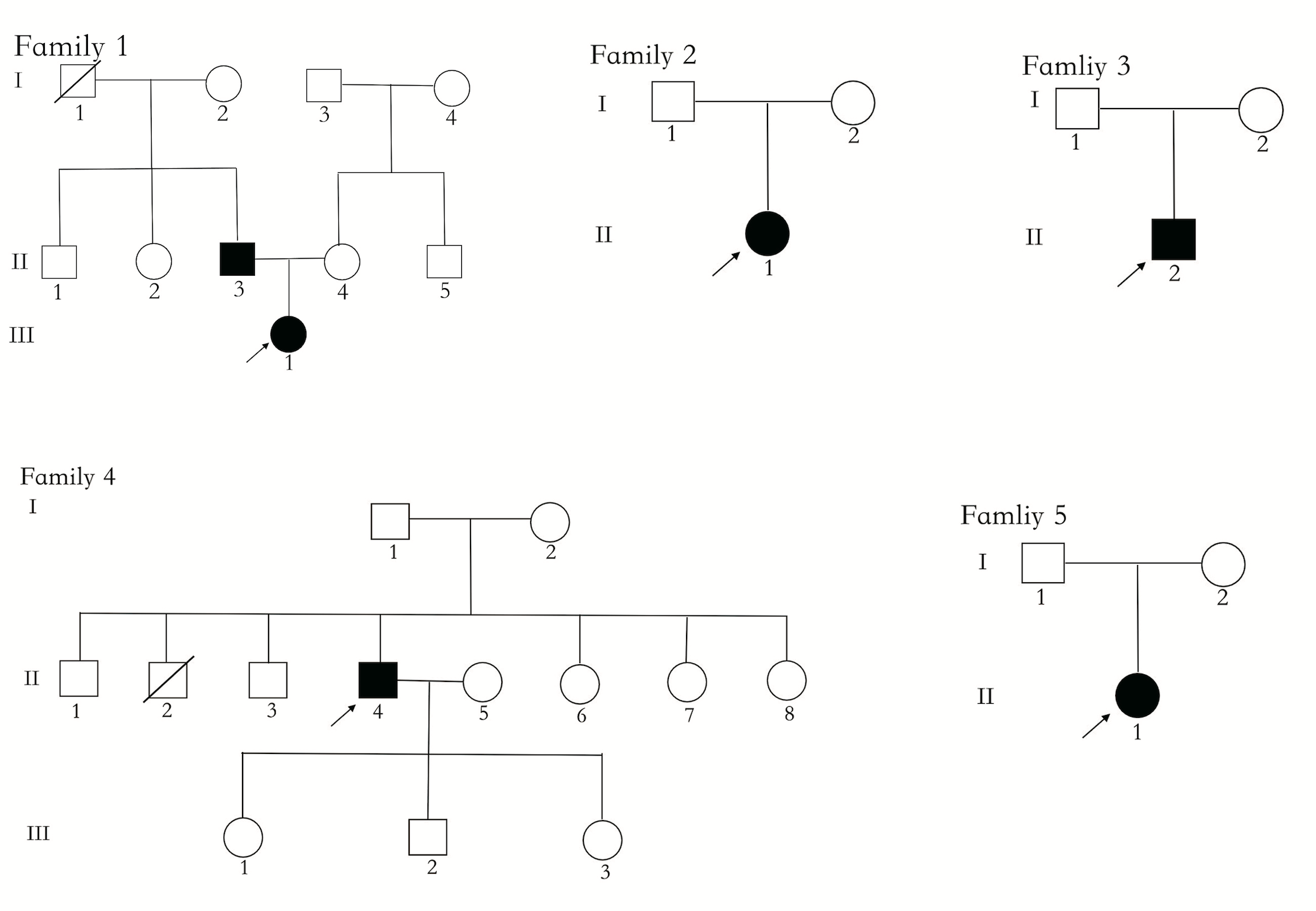

Supplement: Supplementary Figure 1 — Family pedigree of the five probands with NF. “⏹”: affected male individual. “⏺”: affected female individual. “◻”: unaffected male individual. “⭘”: unaffected female individual. “↗”: probands in the family “” dead male individual in the family. [file Image_1.TIFF]

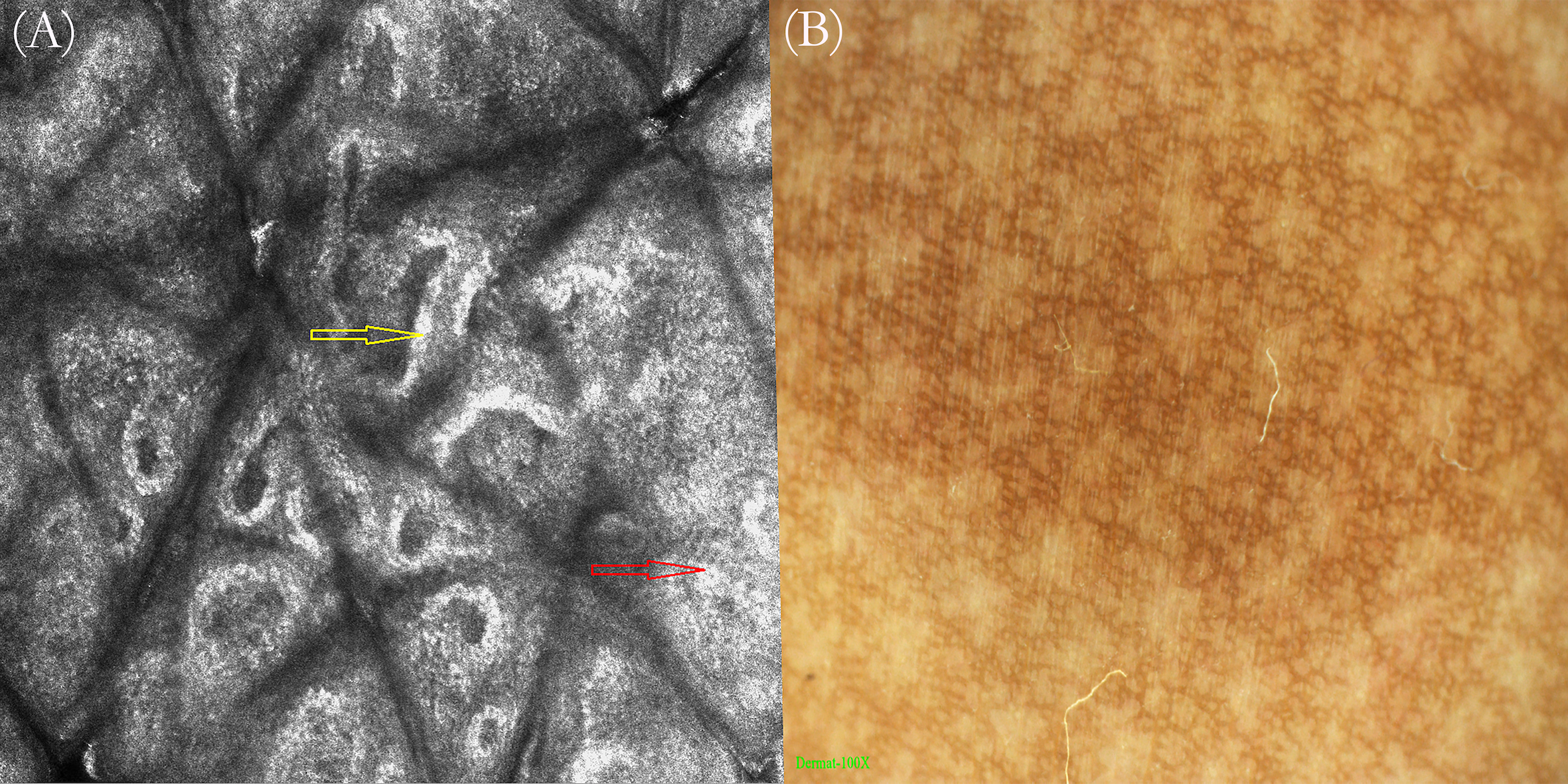

Supplement: Supplementary Figure 2 — Pictures of CALMs by reflectance confocal microscopy and dermoscopy examination. High refractive index particles of different sizes in the superficial dermis (red arrow) significantly increased pigment contents in the stratum layer (yellow arrows) under RCM (A). Regular sepia grid-like pigmentation under a microscope with clear boundaries by dermoscopy (100X) (B). [file Image_2.TIFF]

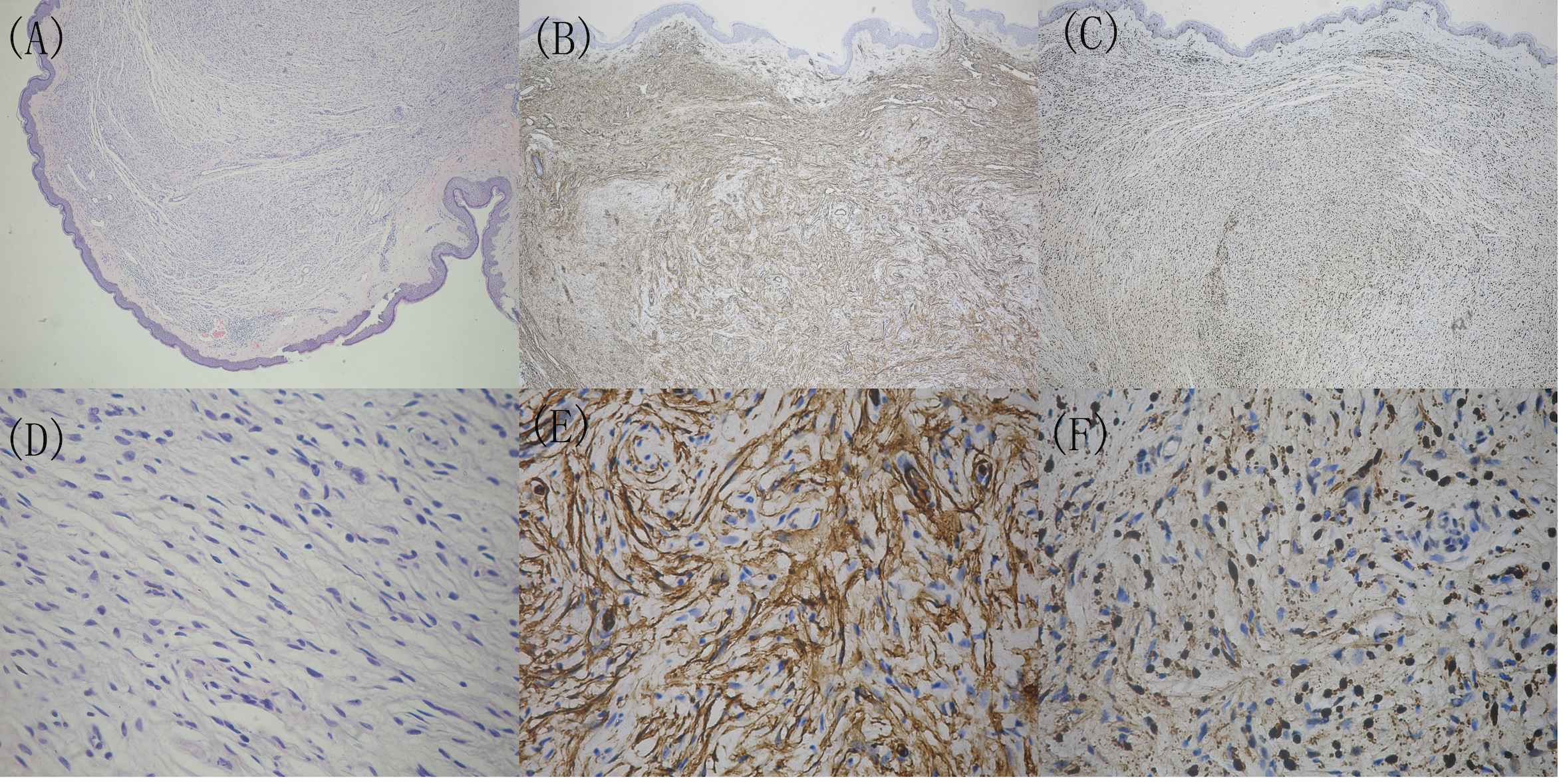

Supplement: Supplementary Figure 3 — Histological and immunohistochemical manifestations of cutaneous neurofibromas in the proband from family 4. A large and isolated nodule in the dermis (A, 10X) and wavy spindle cell changes (D, 40X). Strong and diffuse CD34 staining (B, 10X) and strong CD34 staining in the tumor cell cytoplasm (E, 40X). Scattered S100 staining (C, 10X) and S100 staining in tumor cell nuclei (F, 40X). [file Image_3.TIFF]

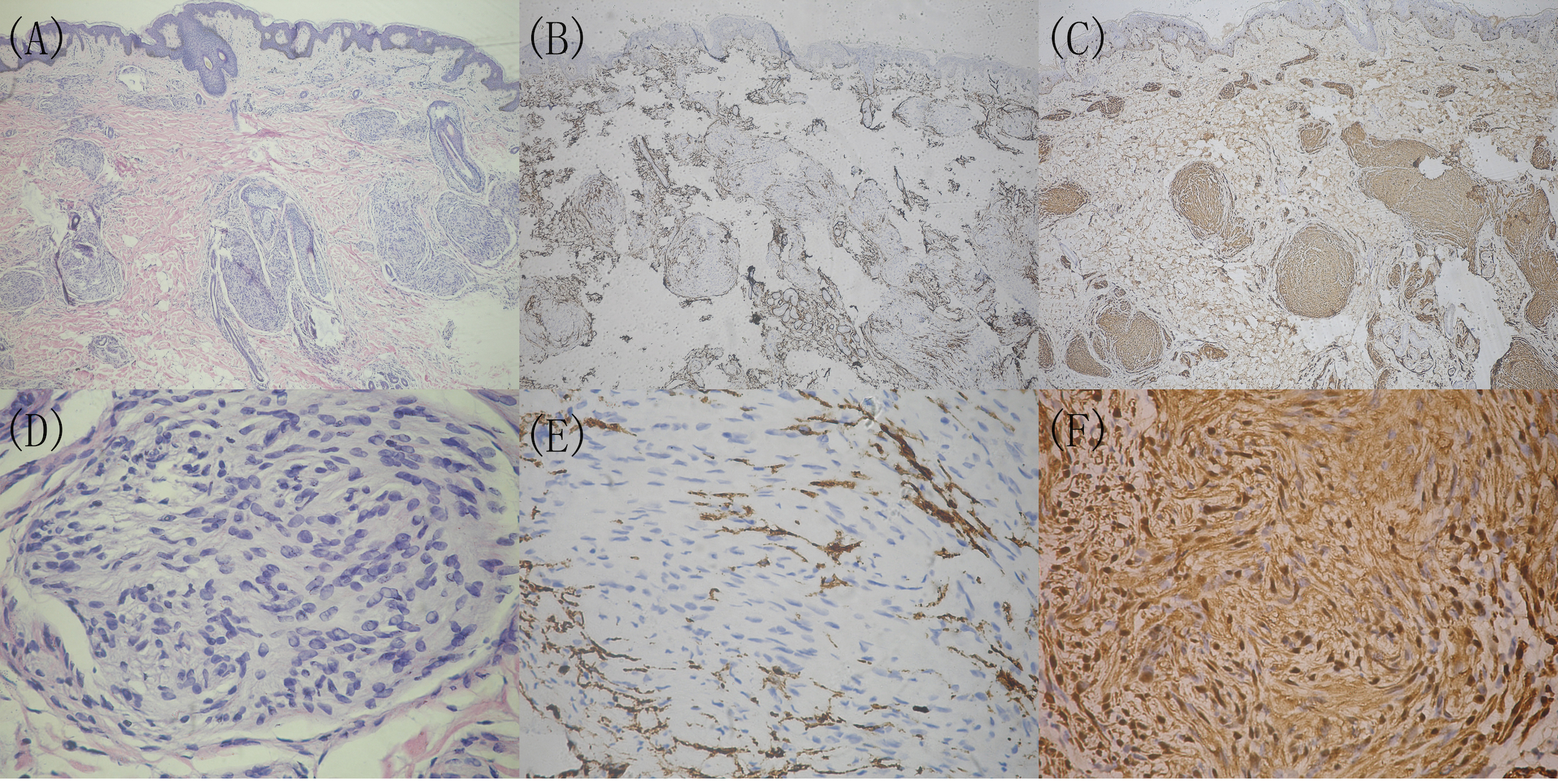

Supplement: Supplementary Figure 4 — Histological and immunohistochemical manifestations of skin plaques in the proband from family 5. The nodules interconnected with each other (A, 10X) and the change in swirly spindle cells (D, 40X). Scattered CD34 staining (B, 10X) and weak CD34 staining in the tumor cell cytoplasm (E, 40X). Strong and diffuse S100 staining (C, 10X) and strong S100 staining in the tumor cell nuclei (F, 40X). [file Image_4.TIFF]
